# Subaltern citizenship: naturalization and belonging for New Russian citizens from Central Asia

**DOI:** 10.1080/13621025.2024.2428667

**Published:** 2024-11-20

**Authors:** Malika Bahovadinova

**Affiliations:** Faculty of Humanities, University of Amsterdam, Amsterdam, The Netherlands

**Keywords:** violence, Russia, Tajikistan, making citizenship, migration, claiming citizenship

## Abstract

This article explores the perils of belonging among recently naturalized citizens in Russia from Tajikistan. As citizenship in Russia becomes accessible to ethnicized migrant workers, it becomes subalternized, a process broader than citizenship’s neoliberalization. A racialized understanding of ethnicity, itself a legacy of the Soviet empire, shapes understandings of political membership in Russia. This has influenced both Russian migration legislation, as well as Russian citizenship, as former migrant workers become Russian citizens en masse. This article also explores how recently naturalized citizens embrace the ambiguity of new political forms of belonging within the deeply charged politics of new citizens’ inclusion. Former migrant workers construe their belonging in both Russia and Tajikistan, claiming their transnational lives and rights to motherland/s as active political agents. Ideas of ‘motherland’, however, are not strictly national or territorial: motherland is understood through localized and spatial experiences and memories. At the same time, former migrant workers’ inclusion as political subjects in Russia has heightened the fragility of Russian citizenship itself, whereby citizenship can now be revoked and annulled with as little as a court order.

## 1. Introduction

Despite its universalist claims, the project of citizenship is structured around the exclusion of non-citizens. The nature of modern citizenship regimes, which emerged in the process of postcolonial separations, has produced not only the independent nation-states of the former colonies but also the hierarchical scales of citizenship (Cooper 2014). Some citizenships became more valued than others, with the figure of a migrant exemplifying various exclusions from the citizenship project. A migrant – a legal status – denotes a non-citizen. Scholarship has long analyzed the coproduction of the experiences of migrants and citizens (Sassen 2002; Harvey 2018). While a citizen implies political membership, a migrant pertains to the separation of rights and legal status. This separation, according to Caglar (2015), has shifted attention to the practices of non-citizens and their social, cultural, and economic participation without the formal legal rights of citizens. This has helped de-center the state-centric approach to citizenship and facilitated attention to everyday citizenship practices and acts (Isin 2008; Caglar 2015; Bloch 2022). These works caution against a strict separation between a citizen and a migrant. In some settings, for example, migrants can vote in local elections, while other citizens with prison experience could be denied political rights (Ruth, Matusitz, and Simi 2017; Finn 2020).

The focus on the blurred boundary between im/migrants and citizens has also elucidated the role of institutions and policy in shaping the limits of citizenship projects. Such works pay attention to the role of law, bureaucracy, and activism, which allows immigrants to become citizens, as well as the considerations of sovereignty that such practices evoke, and the ambiguity of incorporation (Coutin 2007; Friedman 2015). This article is inspired by such attention to the uncomfortable incorporation that occurs when citizenship becomes accessible to marginalized communities. It explores increased rates of naturalization amongst Tajikistani migrant workers in Russia, and the consequent segmentation of the Russian citizenship project as it incorporates racialized migrant workers as citizens. The locally used terms ‘migrocitizen’ or ‘migrant with Russian citizenship’, designated for recently naturalized citizens in Russia, exemplify the perils of such incorporation. In Russia, this segmentation of the citizenship project challenges the overall assumption of citizenship as inherently universalistic.

James Holston suggests the notion of ‘insurgent citizenship’ to analyze the participation of marginalized communities in naturalization processes, which also erodes citizenship projects (Holston 1998). Holston also argues that insurgency becomes part and parcel of this process, meaning the introduction of new sites of political participation and claims to citizenship (Holston, 1998). This article furthers this analysis by focusing on the subaltern group’s *access* to citizenship, and the circumstances of their incorporation as citizens. The term ‘subaltern’ here represents the experiences of extreme forms of marginalization in Russia, where the naturalization process offers the opportunity to bypass harsh migration laws, state violence, and migration policing. I address the colonial legacy of this marginalization and the attendant markedly racialized policing in the context of Russia’s punitive migration legislation that is designed to produce undocumented migrants. My research participants’ decisions to naturalize in Russia were often explained by the freedom it provided from state violence. The large-scale naturalization rates contributed to the respatialization of the Russian citizenship project, and in particular to its segmentization. This segmentation is reflected in the development and the introduction of racialized and differentiated legal provisions specifically for naturalized citizens.

This article also asks how the citizenship project in Russia changes as it accommodates the naturalization of new racialized citizens. What are the legacies and experiences of subalternity amongst Tajikistani citizens in Russia and their relation to naturalization ‘decisions’ in migration? As Russia liberalizes access to citizenship, Tajikistanis have become the major participants in naturalization processes in Russia. Since 2014 there has been a steady increase in naturalization rates amongst Tajikistani citizens in Russia. In 2016, about 30,000 received Russian citizenship, while in 2021 the number increased to 104,000 (Eurasianet 2024). Although application numbers have decreased amongst Tajikistanis in Russia since 2022, they still occupy the first place in terms of naturalization numbers in Russia (Radio Azattyk 2023). To compare, in 2021 31,000 Uzbekistani citizens, who represent the largest share of Central Asian migrant workers in Russia, received Russian citizenship (Najibullah 2022).

This research is also informed by the author’s long-term ethnographic work with migrant workers from Tajikistan in Russia, and with migration officials in Tajikistan (2012–2021). This has provided an important context for the article’s study of increased rates of naturalization in Russia among Tajikistani citizens. The article is based on interviews collected in Tajikistan and Russia in 2019 with Tajikistani citizens who received Russian citizenship. The research team conducted 35 life-story interviews in Tajikistan and Moscow. In this article, I am engaging most directly with the experiences of three individuals who received citizenship in the late 1990s and early 2000s. The selection of earlier experiences of naturalization helps to highlight the postcolonial legacy of ethnic differentiation and experiences with Russia’s evolving migration legislation that is turning towards penal and financialized controls.

## 2. Subaltern citizenship

The concept of a ‘subaltern’ subject is an ongoing academic debate. Antonia Navarro Tejero, for example, has engaged with Fernando Coronil’s definition of a subaltern as neither a sovereign nor fully dominated subject. Subalternity emerges as a relational quality in the context of uneven power relations. Navarro Tejero goes on to conclude ‘[s]ubalternity defines not being a subject, but a subjected state of being’ (Navarro Tejero 2004, 94). The category of subaltern in this regard is not static: one could be subjected to marginalization in one setting, but not in another. In her famous essay, ‘Can the Subaltern Speak?’, Gayatri Spivak discusses the role of ideology in interjecting a marginalized subject with desires and choices written and articulated for her by imperialism and patriarchy. Subalternity, accordingly, as a condition both emerges and is silenced in political, colonial, gendered, economic, and social margins. Spivak asks the reader to explore the politics of the construction of the Other. She suggests looking at the various methods employed in producing the Other: ‘[I]t is also that, in the constitution of that Other of Europe, great care was taken to obliterate the textual ingredients with which such a subject could cathect, could occupy (invest?) its itinerary – not only by ideological and scientific production but also by the institution of the law’ (Spivak 2010, 35). Spivak calls readers’ attention to the politics of representation of a subaltern, how the Other is constituted in text, language, and also the law. The epistemic violence in defining a colonial subject is the production of the Other. I build upon this focus to consider how the representation of a social group can produce legal norms and other forms of marginalization.

Spivak’s location of the subaltern in the margins, but at the same time as an integral subject of economic, legal, and political processes, helps me to elucidate, in this case, subalternity as a condition emerging in the context of extreme marginalization and migrants’ representation as ethnic and racialized outsiders. This condition is powerful not only to nudge naturalization decisions but also to produce a separate legal domain for new citizens. Tajikistani migrant workers in Russia often occupy the lowest niches in the Russian labor market. The words ‘Tajik’ and ‘migrant’ are used synonymously in Russia, despite the factual heterogeneity of migrant workers (Grigorichev 2012); Tajiks, in fact, are not even the largest subgroup of migrant workers. This equation of a Tajik and a migrant in Russia shows that Tajikistani citizens are treated as the furthest extreme of an ethnicized Other amongst the Russian migrant population (Bahovadinova 2025).

My use of ‘subaltern citizenship’ points to relational aspects between the project of citizenship and the scope of naturalization rates amongst Tajikistani citizens in Russia. Vazira Zamindar’s historical account of citizenship regimes in Pakistan and India provides methodological insight into this case of institutional and postcolonial unraveling. Zamindar discusses the long process of postcolonial partition between Pakistan and India, embedded in bureaucratic instruments that produced refugees, citizens, and institutional violence. Partitioning created not only the bifurcation between citizens and aliens but also statist ideas of ‘loyalty and legitimacy’ which marked ‘suspect/disloyal citizens from putatively natural ones’ (Zamindar 2007, 11). In this process, citizenship provisions were centered on the definition of ‘migration’ in India and Pakistan (Ibid., 12). Methodologically, I am drawing on two elements of Zamindar’s analysis. First, I am working with the so-called ‘first’ generation of naturalized citizens from Tajikistan in Russia, some of whom were born and raised as Soviet citizens.^1^ The attention to the earlier instances of naturalization allows me to trace how changes in migration legislation in Russia forcefully reinscribed colonial assumptions of ethnic difference, which were to be policed through an application of laws and police violence – the form of post-Soviet separations. Looking at the Soviet legacy also allows me to draw on the ethnic hierarchies that were embedded in the process of Soviet state making, and which became particularly salient as Central Asia became a major labor exporter to Russia. I also focus on earlier instances of naturalization to explore the gendered experience of police violence in migration. All cases will touch upon various forms of violence in migration and policing, which overwhelmingly target Central Asian men.

Second, I draw on Zamindar’s attention to the bureaucratic legacy of nationalizing states in producing internal differentiation within the population based on presumed ideas of ‘loyalty and legitimacy’ (Zamindar 2007). Ethnic differentiation is important for considerations of ‘legitimacy’. In this postcolonial space, the large levels of naturalization amongst Tajikistani citizens in Russia has contributed to changes in the Russian citizenship project and its gradual incorporation of differentiated belonging. This form of differentiated provisions of citizenship is affected by considerations of ‘loyalty and legitimacy’ in claims to belonging. It highlights the ambiguity in the incorporation of needed but undesired ethnically/religiously different citizens. This ambiguity is bureaucratically productive, expanding the subaltern elements of citizenship laws, and in particular the segmentization of citizenship provisions for naturalized citizens.

Susan Koshy’s critique of the preoccupation with Western influence on the rest of the world, leading to the silencing of the effects of globalization is also of particular interest. Koshy’s term ‘minor transnationalism’ helps me to explore ‘the differential erosions of boundaries to the movement of people and goods’ (Koshy 2005, 116). This article addresses the uneven analysis of citizenship, where citizenship regimes and migration laws define the parameters of non/participation. In this analysis, I approach citizenship as a historical and political project enmeshed in relationships that do not only encompass rights, practices, and legal obligations but also relational dynamics based on assumed ideas of ‘legitimacy and loyalty’. Moreover, it addresses the role of the colonial legacy of ethnic differentiation and the affective registers Russia elicits to this day among recently naturalized new citizens (Reeves 2014).

## 3. Russia’s migration laws and citizenship opportunities

Citizens of the Central Asian states of Tajikistan, Kyrgyzstan, and Uzbekistan represent a large proportion of the migrant workers in Russia. After the disintegration of the Soviet Union, these countries became major labor providers for Russia’s construction and service sectors, with mobility abetted by the visa-free travel regime between the countries of the Commonwealth of Independent States (an intergovernmental organization established after the Soviet collapse). This visa-free mobility facilitated and eased increasing labor migration to Russia from former Soviet Central Asian republics, which were grappling with the legacies of underdevelopment and the abrupt and uncoordinated severance of Soviet infrastructure and economic ties that had led to plummeting economic recession (Scarborough 2023).

Visa-free mobility, however, does not necessarily entail freedom of movement. As mobility for work to Russia grew from the former Soviet Republics, so did the infrastructure of policing and internal controls, indicating a gradual shift towards a focus on the figure of an ‘illegal’ (Bahovadinova 2016). Russian migration law and the frequent changes made to it are used to control and contain migrant workers (Schenk 2018). In 2012, moreover, Russia’s migration law started to change drastically with the introduction of digital technology, in particular with the implementation of a ‘system of electronic accounting of foreign citizens’. This has drastically changed the circumstances of access to the Russian labor market for millions of migrant workers. The new system represented a large-scale deportation infrastructure, with workers increasingly finding themselves removed from Russia, their passports linked to re-entry bans, and unable to return to Russia (Bahovadinova 2016; Reeves 2017). In addition, as of 2014, Russia introduced a series of changes to its migration legislation. Migrant workers were now required to pass Russian language, history, and legal knowledge tests to receive a *patent*, a paid work permit that gave them the right to work in Russia. These amendments sharply increased the costs of mobility to Russia, with the *patent’s* monthly fee generally amounting to a significant proportion of an individual’s earned income. This has affected the way access to Russia is valued and practiced in the context of labor mobility. One of the reactions has been migrant workers’ shift towards receiving Russian citizenship through a variety of official and semi-legal channels, access to which is complex, contingent, and costly (Borisova 2020).

Following these changes, migrant workers have started to effectively use the legal channels available to them, including Russia’s laws on naturalization, to access Russian citizenship. In 2022 alone, 174,000 Tajikistani citizens received Russian citizenship (Asia-Plus 2023a). This was facilitated by the overall liberalization of Russia’s citizenship legislation and the expansion of the government’s program for the ‘resettlement of compatriots’. This was a program intended to increase the Russian population by encouraging the ‘return migration’ of expatriate Russians. According to the programme, knowledge of Russian, affinity with Russian culture and traditions, and formerly held Soviet citizenship now qualified individuals to acquire Russian citizenship (Woodard 2022). In 2023, moreover, Russia’s President, Vladimir Putin, signed Order № 350, which introduced additional changes to the previous year’s Order ‘On fast-track acceptance into the citizenship of the Russian Federation for foreign citizens and persons without citizenship who have signed military contracts’. Both the updated and earlier versions of this Order provided any person who had served in Russia’s ‘military operation’ in Ukraine for at least a year the right to apply for and receive Russian citizenship. Before 2022, by contrast, a person had to conduct military service for three to five years to qualify. The family members of such applicants were also now qualified to receive fast-track citizenship (Rossiiskaia Federatsiia 2023a). The same new version of the law now also theoretically allowed for Russian citizenship to be acquired at birth by anyone born in the Russian Federation, thus adopting the legal standard of *jus soli* over the previous principle of *jus sanguinis*. Tajikistani citizens have become major participants in this liberalizing citizenship infrastructure – but not without consequences.

Since 2017, amendments have been introduced to the Russian Law on Citizenship. Amongst these in 2017 was the new Article 11.1, which now required naturalized citizens to give an oath of loyalty to the Russian Federation when they received their new passports. Article 22 was also added to the Law on Citizenship, which stipulated the possibility of revoking a naturalized person’s Russian citizenship if a court determined that the original provision of citizenship was issued based on counterfeit documents or if an applicant refused to read out the oath to the Russian Federation (Rossiiskaia Federatsiia 2017). In October 2023, moreover, a new Law on Russian Citizenship came into force, which created further grounds to revoke citizenship. Those refusing to serve in combat could now be stripped of Russian citizenship, as well as those who were found to have discredited the army of the Russian Federation. These provisions, it is worth noting, were not meant to be applied to those born with Russian citizenship, but instead could only be applied to those who had been naturalized (Rossiiskaia Federatsiia 2023b, 21–23; Minushkina et al. 2023; Tass 2023).

The large-scale turn to Russian citizenship amongst Tajikistani citizens had been facilitated by changes to citizenship laws in Russia. This, however, had also been precipitated by earlier naturalization rates amongst migrant workers in the early 2000s. The broader wave of naturalization had also been institutionally and legally backed since the mid-1990s: Tajikistan and Russia signed a Law on Dual Citizenship in 1995, which has since allowed citizens of both countries to retain their initial citizenship in the case of naturalization into either Tajik or Russian citizenship.^2^ Liberal citizenship laws alone, however, do not address the circumstances and decisions of naturalization among migrant workers in Russia. The turn to citizenship was also addressing expanding postcolonial legal and institutional separations, processes also observed in other postcolonial contexts (Cooper 2014; Zamindar 2007). The following sections of this article consider three personal stories of naturalization, considering how these factors come together in practice. The first case of naturalization in Russia particularly exemplifies the colonial legacy of incorporation, and the process of its erosion – or at least its segmentation – through the development of different legal provisions that target naturalized citizens.

### 3.1. Karomat’s removal: ethnicity and the perils of Russian citizenship

Karomat Sharipov was well known in Russia and Tajikistan. He was the head of the ‘Tajik Labour Migrants’ diaspora organization in Moscow and he had a scandalous reputation, ranging from rumours of having committed murder or racketeering to questions about his treatment of Central Asian workers in Russia (Asia-Plus 2017). The organization that he oversaw also evoked mixed feelings among migration officials in Tajikistan, as they believed diaspora organizations were inherently untrustworthy and benefiting financially from migrant workers’ earnings.

In December 2017, Karomat was deported to Tajikistan, a country of which he and his lawyers claimed he had never been a citizen. Although born in the Faizabad District of Soviet Tajikistan, and ethnically Tajik, he was not a Tajikistani. He had grown up in Soviet Tajikistan, and after graduating from a local school, he enrolled in the Simferopol Military Academy in Russia, where he graduated as an officer in 1984 and served in the Soviet Army in Kazakhstan and West Germany. After the removal of Soviet troops from Germany, he was sent to Tajikistan, where, in 1993, Karomat joined the 201st Russian Military Division, a Russian garrison based in Tajikistan. From 1996 to 2000 he was stationed in Moscow with the division, and then retired from the military in 2001. Afterwards, he established his diaspora organization (Zakhvatov 2016). As the head of this organization, he spent much of his time writing letters to the Russian prosecutor’s office about the torture of Central Asians in Russian prisons or the leniency of verdicts in cases where Central Asians had been the victims of far-right violence; the organization was also engaged in labor dispute cases (Tadzhikskie Trudovye Migranty 2001; Sharipov 2009). According to his lawyers, in the early 1990s, Karomat had exchanged his *Soviet* passport for a Russian one, which his service in the 201st Division legally allowed him to do.

In 2016, the Russian High Court announced its decision to liquidate Karomat’s organization, the ‘Tajik Labour Migrants’. In 2017, moreover, Karomat’s Russian passport was confiscated, and he was charged with falsifying the documents he had used to receive Russian citizenship. The court found Karomat guilty of violating article 18.8 of section 3.1 of the Administrative Code of the Russian Federation, which covered violations by foreigners or persons without citizenship of the rules of entry and stay in the Russian Federation (Radio Ozody 2017). Karomat argued that his deportation was politically motivated and related to a case he was working on against a Russian firm that had failed to pay workers’ salaries. When Karomat tried to sue the officials in charge, he was issued an order of administrative removal from Russia and was subsequently escorted to the airport and deported to Tajikistan.

Karomat’s partner and children were Russian citizens, which, according to Russian law, was sufficient to remove his re-entry ban by judicial order – in other words, Karomat should have been allowed to stay in Russia. In this case, however, it did not help. Karomat was sent to Tajikistan. The Tajik Consular Office in Moscow issued the court a paper confirming that he was a citizen of Tajikistan. When his lawyer asked the judge what his wife and children, Russian citizens, should do in this situation, the judge suggested that they could follow Karomat to their ‘historical motherland’ (*istoricheskaia rodina*).

An ethnic Tajik but not a Tajikistani, Karomat’s removal from Russia was indicative of the limits of Russian citizenship for ethnically marked citizens in Russia. The judge’s remark about ‘historic motherland’ points to the relationship between ethnicity and belonging as construed in official Russian narratives. The legacy of Soviet definitions of ethnicity and the idea of belonging *to* a place is important for the analysis of citizenship and the limits of belonging in Russia. In her work on borders and peoples’ experiences with borders, Reeves (2014, 57) suggests critically exploring the ‘ethno-spatial fix’ – that is, the idea of a natural ‘fit’ between people, culture, and a given territory. Ethnicity in Soviet and post-Soviet analysis became biologized, linked to land, and discussed as the true and ultimate marker of identity (Reeves 2005). This strict ethnic fixing supports the notion that ‘blood’ (i.e. ethnic markers and linguistic and cultural practices) and territory overlap to produce a nationally bounded polity of citizens (also see Abashin 2004; Hirsch 2005). The legal provision allowing for the stripping citizenship introduced in the law ‘On Citizenship’ in 2014 exponentially expanded the number of citizens who potentially could now have their citizenship revoked following the aforementioned Article 22. Liberalization of citizenship laws was circumscribed with considerations of legitimacy and loyalty, in this context drawn across ethnic differences. The ethnic difference ascribed to Karomat produces his representation as belonging ultimately elsewhere, further entrenching his marginalization in Russia. The Soviet legacy of ethnic differentiation spoke for Karomat louder than his claims to belong in Russia.

As ethnicized groups have increasingly turned to naturalization in Russia, the Law on Citizenship was changed to ease their removal in pertinent cases. In their analysis of the modern slippery slope of citizenship, Isin and Nyers (2014) propose to think about the situation through Derrida’s concept of ‘erasure’. For Derrida, ‘to write “under erasure” is to write and delete simultaneously, to script and cross out, to present and bracket, create and destroy. Erasure allows positing something affirmatively and yet remaining skeptical … . In a sense erasure posits a different temporality “no longer” and not-yet’ (Ibid, 6). This anxiety among recently naturalized citizens reflects Karomat’s worry not to brag about Russian citizenship because ‘one always stays Tajik for “them”’ (Asia-Plus 2017). Karomat understood this dynamic quite well, having lived and worked in Russia since the 1990s. As a Russian soldier who had ‘consumed’ the Russian state through his provisions and pay (see Reeves 2012), he knew well enough that his ethnic markings as a ‘Tajik’ would ultimately trump any other affective connections with Russia. These ethnic markers are attributed not only to ethnicity, culture, or religion but also to phenotype and other external features, as I will explore in the further cases of Ravshan and Hafiz.^3^

### 3.2. Ravshan: subalternity in Russia

Hauling a heavy cart loaded with metal and rubber, Ravshan was repeating to himself the Russian words he had learned the night before from the beat-up Russian-Tajik dictionary he had bought during his last trip home to a village in the North of Tajikistan. The heavy load was hard to pull, but every day Ravshan repeated and memorized the three Russian words a day he had committed himself to learn. He needed to know Russian. The contract and paperwork for his daily work volumes were in Russian. To calculate his hours and workload, the proper terminology was important. A mistake could lead to deducted pay. For a year and a half, Ravshan hauled this cart and repeated words, toiling at a job that his father had helped him to secure in the same auto parts shop in Moscow where he worked.

Ravshan had grown up in a village in the South of Tajikistan with his two younger brothers. Their father was once a gym teacher at a local school, while their mother worked in the local kolkhoz. His father first left for Russia, Ravshan explained, when increasing financial demands outgrew the modest salaries his parents earned. His father would come home to the village from Moscow once a year. In Moscow, he spent his months toiling like Ravshan: heavy lifting in the same auto parts shop for a decade. Ravshan noticed his father’s health deteriorate when he was in the 10^th^ grade at school. As he watched his father weaken because of his heavy work (*korash vazmin bud*), he made the ‘decision’ to join him. The ‘choice’ of going to Russia, however, was structured with societal and gendered expectations on him as the eldest son responsible for helping the family. He told his father, ‘I also will go to Russia’ (*hai man meravam ba Rasia*). His father agreed, and after Ravshan graduated from school he joined his father in Moscow.

Hauling the cart for a year and a half, Ravshan was increasingly frustrated with the cart’s weight. He worked two shifts, his lunch break was 15 minutes long, and he did not have much rest. He wanted to escape the job: his patience with the cart was running low. Hauling it, he dreamt about trade, about selling produce at the market:

Du smena telezhka ruzi daroz kashola mekardam. Yagon hele odih, vai nabud. Hai Hudoba shukr kor kardem, darvakht hamin niyat mekardam ki, Hudo agar hohad ravam Rasiaba yakta tochka mekushoyam, e yak savdo mekunam.

I hauled that cart two shifts in a row every day. There was no sort of rest. But thanks to God we had work; with time I started to plan that, God willing, I would come back to Russia and open a little shop or get involved with trade.

Before telling his father he was done working with the cart, Ravshan explored the possibility of working in a market on the outskirts of Moscow where he had a few acquaintances from his village. A shop at the market was also about to go on sale. When he returned to the auto parts shop, he told his father about his decision: he wanted to work in trade. At the time, he already had a temporary residency, the first step required towards receiving Russian citizenship.

When Ravshan first arrived in Russia, his uncle, who was living in a small city in Western Russia, had already suggested that he apply for citizenship. Now the decision was quick. On a trip to Tajikistan, Ravshan’s father collected and brought back all the documents needed for the application. Ravshan’s uncle’s ‘friend’, a broker, managed the processes, for which Ravshan was expected to pay a significant amount. Ravshan went twice to stay with his uncle. First to receive his permanent residency and the second time to receive his passport.^4^

The broker charged a hefty amount at the time for facilitating this relatively easy access to naturalization. Ravshan, however, saw the citizenship ‘pay off’ already the year he received it, in the mid-2010s. ‘When I received my citizenship, our director took out a law and said that from that year on we were to receive our work permits ourselves. That very year I saved (*vygrish kardam*) 25000 rubbles … that year I was very glad’. Ravshan was referring to the gradual shift of legal responsibility for migrant workers away from a business or enterprise (a remnant of Soviet practice) towards individual responsibility placed on workers to secure their legal stay in Russia.^5^

In general, Ravshan was satisfied that he had become a citizen in Russia. This is how he explained his feelings:

Some are saying that I sold my motherland (tu vatanfurushi kardi), but look, out of the 7–8 years that have passed since I arrived here, I spent 6 here and 1 at home. Therefore, vatanfurushi does not make sense (inja ni pri chyom). In addition, there are such issues as well as not being harassed on streets (kucha gam nadidand) and being left alone to work in one place. Here I am selling at the market. If not for citizenship, I would not have been able to do it. I would have to get a work permit (patent) and still I wouldn’t be selling.

Ravshan was referring to the legal provisions that were introduced in 2006 that created an ‘acceptable quota’ of foreign workers ‘used in various sectors of the economy’ (Vitkovskaia 2009). These measures targeted specific economic sectors, including retail trade. Migrant workers with work permits were no longer allowed to sell pharmaceuticals and alcoholic beverages, and from 2007 their participation in trade was prohibited altogether in markets and shops (Vitkovskaia 2009). These laws produced a racialized separation where migrants should not be in contact with Russian citizens (Meduza 2024).

As a citizen of Russia, however, Ravshan could buy and sell produce at the market – and without having to pay monthly fees for a work permit. Although he discussed the financial benefits accruing from his new citizenship, the materiality and value it produced were not just given or expected, they were gradually engendered by the law and the broader legal landscape, which had become increasingly punitive in nature, specifically targeting citizens from the former periphery (Reeves 2014). This was the process of separation. The types of jobs in these separations for people like Ravshan were also physically demanding, exhausting, and ideally invisible (Isabaeva 2013). Ravshan’s father, a schoolteacher in Tajikistan, had only been able to find a job as a hauler in Russia.

Gradual changes towards racialization and financialization of migration laws pushed workers to apply for Russian citizenship (also see Sadiq 2008; Ruget and Usmanalieva 2010; Aptekar 2016). Confronted with changes in legislation, naturalization in this regard was not just a matter of careful consideration of costs and benefits, or the instrumental consideration of accessing the labor market, however, but also to reduce violence in migration. Stop and frisk procedures remain part and parcel of the infrastructure of policing in Russia (Reeves 2015; Bahovadinova 2025). Often, detentions and bribery accompany these practices. Ravshan specifically mentioned ‘being left alone on streets’ as an integral benefit of Russian citizenship.

Ravshan was also very careful in his response to the question about the meaning of this new citizenship for him. He said: ‘I don’t brag about it. I am always a citizen of Tajikistan (*sharhvandi Tojikiston*), and always will stay a citizen of Tajikistan. I have never said anywhere that I am a Russian citizen (*ya grazdanin Rossii naguftam*). I say that I *have* citizenship’.

Interviewer: Why don’t you say that you are a citizen of Russia?

‘Ravshan: This would be *vatanfurushi* (selling the motherland). I was a citizen of Tajikistan, and I am. And I don’t mean citizenship, like how I am a citizen of Russia… This is the place of my birth, it is the place of [my] water and air (*obu havosh*), it is the place where I grew up, in those places [pause] … . Such a place, you cannot replace it with paper and money. If they catch me [in Russia], the police, I never say I am a citizen of Russia. I say: I forgot my documents at home (*Man meguyam dokumenti doma zabil*)’. Even when discussing his affective relationship to his new citizenship, Ravshan’s reference to documents emphasizes the centrality of the documentary regime in policing migrant workers in Russia (Reeves 2015).^6^

This careful avoidance of citizenship claim-making can also be analyzed in the wider (and global) field of racialized treatment of minorities. As Su’ad Abdul Khabeer (2017, 113) noted in his analysis of citizenship amongst Muslim American youth, for some of his interlocutors ‘affective citizenship’ was not a possibility, because the idea of being an American was deeply intertwined not only with ‘whiteness’, but also with the oppression of minority groups. In certain circumstances, Khabeer (114) argues, citizenship claim-making becomes untenable for some individuals. Ravshan also noted on his list of ‘benefits’ that he was no longer harassed on the streets, referencing the Russian police’s stop-and-frisk practice. From the quote above, it is clear that this did not stop after Ravshan received citizenship, although state violence was significantly reduced as a result. Ravshan was deeply ambiguous about his Russian citizenship, locating it in the realm of paper and not affect. As Khabeer (108) succinctly observed, documents alone do not produce a ‘legitimate citizen’ – they need to be complemented with racialized and gendered expectations of normativity.

Ravshan’s ideas about belonging in Russia or Tajikistan do not refer to forms of diasporic citizenship based on a concept of a ‘motherland’, but instead reflect his more personal response to becoming a political subject in a marginalizing society. Racialization, which found its way into legal provisions dictating that migrant workers were not supposed to be dealing with the ‘public’ (as public transport drivers and working in trade), expanded the conditions of migrant workers’ subalternity. Naturalization, in this reading, is a process of ‘negotiated presence’ that is found in the gradual shift of post-Soviet migration law towards a financialized and penalizing mode of ‘inclusion’ for Central Asians as ‘migrants’. Ravshan was anxious to not make citizenship claims in Russia as a citizen of Russia: this would have been ‘*vatanfurushi’*, he said, insisting instead on a mode of document-based civic citizenship and his remaining claim to difference (Rosaldo 1994). At the same time, Ravshan was still comfortable asserting his rights as a citizen of Russia to stand and sell in the market, reflecting the changing nature of that institution of citizenship and his place within it as an outsider. On this background, the final case study, that of Hafiz, provides a poignant depiction of the value of political membership and the willingness to fight for a new motherland if such need arises, even as recently naturalized citizens understand the limits of their incorporation.

### 3.3. Hafiz, the law on dual citizenship, and the new life in Russia

‘I am, hmm, how to say, a patriot [of Tajikistan]. For a while, I did not want to receive Russian citizenship not to betray my country. They were telling me that a document (*spravka*) about the renunciation of my [Tajik] citizenship was required [to receive Russian citizenship]. But when I learned that when you receive Russian citizenship, your second citizenship stays, this is what motivated me to seek it’. Hafiz, a man now in his mid-40s, first arrived in Moscow in 1998 after graduating from university in Dushanbe. He lived and studied in Dushanbe at the start of the Tajik Civil War (formally 1992–1997) as a Pamiri, an ethnic and religious minority from the east of Tajikistan. Because of his ethnicity, which was associated with one side of the conflict, Hafiz was beaten and threatened by other students (see Scarborough 2023 on the violence of the Tajik Civil War). He became a ‘student-refugee’, as he self-described himself, and in 1992 he fled from Dushanbe to the Pamirs, an autonomous region of Tajikistan, which remains today severely suppressed by the central government. Frustrated about dropping out from university, he eventually returned to Dushanbe to reinstate as a student and complete his studies, despite the ongoing civil war and the targeting of Pamiris by pro-government troops.

Hafiz received his diploma in History and State Law in 1997. In 1998, he left what he recalled as ‘sunny Dushanbe’ and arrived in rainy Moscow. He was then summarily ‘forgotten’ by his relatives, who had been meant to meet him at the airport. Picked up by another Pamiri man who took pity on him, he was taken to a city outside Moscow and offered a job as a loader. Although he was frustrated at having to work in construction with a university diploma, he nevertheless stayed put and gradually brought his brothers to join him in Russia. Speaking in fluent Russian, Hafiz notes that he learned the language in his local school in Tajikistan from a Russian teacher who had transferred to their rural village from then-Soviet Russia.

Hafiz later moved to Moscow for work, where he also started to volunteer with a Pamiri diaspora organization in early 2003. It was then that he started to think about citizenship:

When I was living in Moscow and working with the diaspora organization, there were constant checks of my documents on the streets. I was also beaten. Once I had to be collected from the police station where I was taken. They just came to my home and took documents and me to the station. There were such moments; in the metro, I was also stopped often.

For Hafiz, the initial reason for acquiring citizenship was largely related to the police violence he experienced in Moscow. This coincided with the increased incidence of naturalization amongst his acquaintances who were also supported and encouraged by the head of the Pamiri diaspora organization. Helping other men with money and advice, he suggested that they consider purchasing real estate in particular locations in Russia as the first step to naturalization. The basic idea was that by owning a home, a foreign citizen could claim permanent legal registration at a physical address. This would give legal access to the temporary residency permit that precedes permanent residency and citizenship. At first, though, Hafiz had only planned on earning enough money to return and settle down back in Tajikistan. He had not been planning on naturalization in Russia:

I did not worry. But when I started to face difficulties, and saw how others, who had citizenship … they were more protected, it seemed like they did not have problems. They were not stopped, and if they were stopped, they had a green light. They also had a green light for finding a job … .Life has become calmer, you know you won’t be stopped by police, you are a citizen of Russia (ty grazdanin Rossii), when you see the police, you walk assuredly (idesh uverennii takoi). When you don’t have a normal document, you show it, they guess it. but now you don’t even think about it.

When I interviewed Hafiz in 2019, he was not planning on returning to Tajikistan. As an older brother, he had brought his younger brothers to Russia, and they had settled in Moscow and the surrounding region with their families; their children were already in Russian schools. Space for him had grown shorter and more malleable: ‘Today’, he said, ‘you are in Russia, tomorrow in Tajikistan or in Europe. From here to Dushanbe, it is a short distance (*eto pustiaki*)’. But if he had to choose, he said, he would keep his Russian citizenship: ‘To be a Russian citizen – this is to be loyal to your motherland, to follow its laws, to be part of civil society and serve Russia. To be a patriot of Russia’. At the same time, Hafiz was acutely aware of the limited nature of his belonging in Russia. He feels as though he is an equal citizen juridically, but not socially. Legally, he and his family are on equal ground: his children all have proper registrations in Russia, they have access to schools, and his wife receives state maternity benefits. Yet he feels discriminated against when looking for jobs, and he is often told by people on the streets to ‘go to his home’ – he cannot escape the feeling of not being local. ‘But on the state level, I am an equal citizen of Russia’, he explained about his sense of being a naturalized citizen. It is this legal equality that he attributed to the merits of the state and the need to protect that state if he would be mobilized.

## 4. Conclusion

Living and working in a highly charged racialized environment, Tajikistani citizens in Russia employed the naturalization process to significantly reduce the field of violence in migration. For Hafiz, a passport carried quite literally bodily consequences. As he noted, once he became a Russian citizen he was walking calmly and poised on the streets. Hafiz saw his naturalization as the moment of transition to a calm and legal equality – even as the institution of Russian citizenship itself became shakier and more tenuous as more people like him became naturalized in Russia. The embodied assurance that the Russian passport gave him, however, was rooted in and rested upon the racialized treatment of migrant workers. When police raids occur in Russia in the places where migrant workers are employed, the first question asked of detained crowds of workers is: ‘Are there any citizens of the Russian Federation here?’ (Bahovadinova 2025).

Karomat’s experience exemplified the space of ambiguity in the post-Soviet legacies of postcolonial separations. He moved from Soviet citizenship to Russian citizenship, maintaining, however, and admitting his difference as ethnically Tajik. His shaky legitimacy as a Russian citizen was premised on considerations of ultimate loyalty to a supposed ‘ethnic motherland’. This is why Karomiat cautioned against bragging about Russian citizenship, because for ‘them’, he noted, ‘you are always a Tajik’. The Soviet legacy of ethnic belonging helps to explain why the Tajik Consulate issued him a certificate to return ‘home’, and why Tajikistan was the place of his expulsion from Russia. New legal provisions on stripping naturalized citizenship were also introduced after a bombing in the St. Petersburg metro committed by a man from Central Asia, highlighting the social fears associated with ethnically divergent persons in Russia. In practice, moreover, the law was also used to deport and remove activists, including ethnically marked Russian citizens. Another Tajikistani activist who had become a Russian citizen, Izzat Amon, also disappeared from Russia in 2021; he was later found in a jail in Tajikistan, having been charged with fraud – and with his Russian citizenship revoked (for similar cases, see Furstenberg, Lemon, and Heathershaw 2021; Scarborough 2022).^7^

These legal changes reflect two corresponding but contradictory processes: first, the overall legal liberalization of naturalization into Russian citizenship, starting with the expansion of the state program for compatriots over the past decade. This has aligned with broader demographic decline, with natural population loss through mortality only being held at bay through in-migration and naturalization; the Russian state has recognized and incorporated this fact into its migration policy as well (Gontmakher 2022; Vinogradova 2023). The war in Ukraine has further contributed to the introduction of fast-track routes to Russian citizenship through military service. Importantly, however, this opportunity for fast-track citizenship is situated alongside migrant workers’ encouragement to serve in the Russian military in Ukraine. Pamphlets in which young Central Asian men were called to fight for their ‘motherland’ the way their grandfathers fought for the Soviet State in WWII have been distributed in Russia. The legal changes reflect this call for loyalty from new or would-be citizens (Bahovadinova and Borisova 2025).

At the same time, the bond suggested by the new fast-track route carries with it the threat of being undone as a citizen. Citizenship can be revoked if an individual is considered insufficiently loyal or legitimate, considerations that Zamindar also observed in the process of partitioning between India and Pakistan. In the case of migrant workers from Tajikistan to Russia, ethnic and religious differences have produced the circumstances for naturalization decisions (police violence, financialization, and criminalization of migration laws). Ethnically marked citizens, however, are considered neither fully loyal nor fully legitimate. A Russian politician’s question – ‘Where are the Tajik battalions?’ - after war mobilization had started in Russia, poignantly shows the ambiguity of the uncomfortable incorporation of ethnic outsiders (Asia-Plus 2023b). The recent case in which Russian citizenship was revoked from a Tajik for his evasion of military service in the conflict in Ukraine poignantly exemplifies this nexus of loyalty and legitimacy in defining the limits of incorporation of racialized new citizens (Rbc.ru 2024). This article has attempted to explain why politicians in Russia would specifically ask about the lack of ‘Tajik’ fighting units. These representations have led to changes in citizenship laws: those that simultaneously restrict, incite, and also remove those unwilling to fight for Russia.

The expanding legal possibilities for naturalization in Russia have allowed people like Ravshan and Hafiz to bypass migration infrastructure, work in trade, reduce police violence, and lower the costs of migration. At the same time, moreover, the ambiguity expressed among recently naturalized citizens about the risks of ‘selling their motherland’ and their complex affective attachment to Russia and Tajikistan demonstrate a sense of belonging that goes beyond discussions of diasporic citizenship or those directly linked to a single nation-state. The choice to occupy and traverse the indeterminacy of belonging in Russia becomes a process of participating in Russia’s citizenship project. This participation has an important if unexpected influence on the nature of that institution. Russian citizenship, in its attempts to control the influx of newly naturalized citizens and police their connections to the states, has become segmented. It has further expanded through the laws and legal provisions that are developed specifically to control and, if necessary, remove new citizens to their ‘ethnic motherlands’. The development of new legal provisions has extended subalterneity and marginalization from the racialized migrant workers to racialized new citizens.

## Notes

1. In Russia, citizenship decisions became much more diverse after 2015 and incorporated middle and upper classes acquiring Russian citizenship ‘just in case’, for social and economic considerations. Naturalization rates appear to be reducing after 2022 not only because of the war in Ukraine but also after changes to Russian legislation regulating pensions and maternity payments.

2. This helps to explain the relatively high naturalizations rate among citizens of Tajikistan for 2022 if compared to those of Kyrgyzstan (23,000) or Uzbekistan (27,000). In addition, however, citizens of Kyrgyzstan have no longer needed to receive work permits in Russia since Kyrgyzstan joined the Eurasian Economic Union in 2014.

3. Central Asian workers are frequently referred to as ‘blacks’ (*chernie*) in Russia; see Bahovadinova (2016); Kassymbekova (2016). For Tajik responses to racial discrimination and social injustice, see Kalandarov and Molchanova (2021).

4. Ravshan’s original temporary residency permit was from the small city where his uncle was living. He nevertheless worked in Moscow, which was technically a violation in Russia, where residency rules are still strictly applied to Russian citizens and non-citizens alike.

5. Caress Schenk (2018) has called the patent system a practice of ‘state capitalism’.

6. In such instances, the police would detain a person without documents at the police station, where their name and address would be verified in the central database. After several hours, if proved to be citizen, the person would be released from detention. More recently, police have started to carry tablets on which information can be verified. The majority of my respondents, however, were always carrying their passports or copies of passports to show their documentary belonging in the face of potential police checks.

7. This provision became particularly uncomfortable after the annexation of Crimea, when naturalized Slavic citizens began to question the double-edged regime of Russian citizenship.
